# “Setting Them Up for Success”: Including Youth on the Autism Spectrum in 4-H

**DOI:** 10.3389/fpsyt.2022.913600

**Published:** 2022-05-31

**Authors:** Carolyn E. B. McCormick, Veronika Peskova, Abby Morgan, Emily Carlson, Rose A. Mason

**Affiliations:** ^1^Department of Human Development and Family Studies, College of Health and Human Sciences, Purdue University, West Lafayette, IN, United States; ^2^Purdue Extension, Montgomery County, Crawfordsville, IN, United States; ^3^Department of Educational Studies, College of Education, Purdue University, West Lafayette, IN, United States

**Keywords:** autism, extracurricular activities, inclusion, Cooperative Extension, community-based programs

## Abstract

Youth on the autism spectrum often face challenges accessing services in rural communities compared to those who live in higher resource areas. There is a particular need for services that support skills that will help youth transition to adulthood and future employment. 4-H is a national youth development program that is well-positioned to address the needs of youth on the autism spectrum; however, minimal empirical evidence exists about the implementation and effectiveness of inclusive practices in 4-H programs. The goal of this study was to better understand barriers to enrollment and to identify gaps in support for youth on the autism spectrum participating in 4-H. Twenty Extension Educators in Indiana participated in two focus groups. Thematic analysis of focus group transcripts identified barriers to enrollment including awareness of 4-H as an inclusive program and difficulties navigating 4-H culture. Our analysis identified themes related to new training content and delivery including a resource portfolio, communication, individualized accommodations, and working within the existing leadership training structure. Findings support the benefits of 4-H as a program that can promote life skills and personal development for youth on the autism spectrum but also highlight a significant need for additional training opportunities and resources to increase uptake and improve the implementation of inclusive practices.

## Introduction

Youth with autism spectrum disorder (on the autism spectrum) face unique challenges with social communication, including difficulty with initiating and maintaining conversations, understanding social nuances, and developing relationships with peers (1). Youth on the autism spectrum also tend to perseverate or have a limited repertoire of interests, making it more difficult to identify peers with similar interests (2). In adulthood, these differences contribute to higher rates of under and unemployment (3), which impacts the quality of life for adults on the autism spectrum. Stakeholders have highlighted the need for more supports and services with a specific focus on developing life skills that would promote success across the entire lifespan (4, 5). Accessing services that support the development and education of youth on the autism spectrum is often difficult due to cost and availability (6, 7). This is particularly salient for youth who live in rural areas, where issues with the availability of resources are even more pronounced for children on the autism spectrum (8, 9).

One avenue for overcoming resource obstacles, is through the implementation of interventions in natural contexts, capitalizing on well-established social structures and groups (10), such as those provided by community-based programs (11). Community-based programs typically have established peer groups and may also have multiple interest groups which increase the likelihood that involved youth, with and without disabilities, can identify a peer group with common interests. For youth on the autism spectrum, participation provides valuable opportunities to practice communication and social interaction skills, as well as the potential to develop friendships (2). Involvement in organized extracurricular activities has also been associated with less depression and loneliness (12). Young adults on the autism spectrum with a record of high academic achievement have also reported participation in extra-curricular activities as a positive venue to develop both their interests and social skills (13).

One such community program is 4-H https://4-h.org/. 4-H, which stands for Head, Heart, Hands, & Health, is delivered through Cooperative Extension, a community-university partnership that is comprised of more than 100 public universities across the nation. 4-H is the largest youth development program in the United States and serves over 6 million youth with the support of adult volunteers and 4-H professionals. This national program provides local communities with research-based programming that centers around science, healthy living, civic engagement, and agriculture. Programs offered through 4-H focus on using hands-on experiences that empower young people with the practical and life skills they need to be leaders now and in the future. Practical skills and content knowledge can be gained across a wide range of topics such as robotics, animal husbandry, sewing, and photography. Life skills such as leadership, resiliency, and communication, are learned and enhanced through various delivery methods such as camps, community clubs, and in-school and afterschool programs. 4-H programs also offer opportunities for peers to connect and form new friendships. Most importantly for youth on the autism spectrum, 4-H has supporting diversity, equity, and inclusion within its program goals. This means that children with all disabilities, including autism, should have access to all 4-H programming.

Despite the benefits of participation in extracurricular activities including an avenue to develop and explore interests, create new friendships, and learn everyday skills (14), youth with disabilities participate at lower rates than their typically developing peers (15). Additional challenges when it comes to enrollment and participation in extracurricular activities negatively impact the rate of participation for youth with disabilities (15, 16). There are a variety of reasons that may contribute to why many youth with disabilities refrain from engaging in extracurricular activities, including barriers related to awareness, skills, opportunities, and support from teachers, parents, or guardians (17). Countless organizations, including 4-H, have no specific training program for volunteers and employees on how to serve youth with disabilities (18). Investment in inclusive practices in out of school environments increases participation from youth with disabilities (19). Within 4-H, there have been attempts to implement additional programs or resources to specifically address the inclusion of youth with disabilities, however, these have been limited in scope and/or longevity (20–22). Together this indicates a need to increase recruitment and retention efforts for youth on the autism spectrum.

Despite positive attitudes toward 4-H (23) and the national availability of the program, it is unclear why this potentially valuable resource is currently underutilized. As a first step in understanding ways to promote 4-H for youth on the autism spectrum, the goal of this project was to assess the current experiences and practices of Extension Educators to address the following research questions: (1) What are the barriers to enrollment in 4-H for youth on the autism spectrum; (2) What types of training resources are available to Extension Educators within 4-H to create inclusive programming; (3) What supports and strategies do Extension Educators already use to support youth on the autism spectrum within their programs; (4) What challenges do Extension Educators and youth on the autism spectrum face within 4-H; (5) What types of new strategies and resources would help to improve inclusion?

## Methods

We report our methods according to the COREQ criteria (24).

### Participants

The participants for two focus groups were recruited through purposeful sampling via electronic flyers sent through email to the full list of Extension Educators in Indiana and through social media posts. Extension Educators collaborate with the university, organizations, businesses, and schools to provide evidence-based educational programs to their communities. Extension Educators who oversee 4-H Youth Development programs provide leadership to club leaders, counselors, and others who support the implementation of programming throughout the year. They also lead and participate in the implementation of programs. In Indiana, there is one 4-H Youth Development Extension Educator in each of the 92 counties. All 20 participants (5 male, 15 female) were current Extension Educators in Indiana who worked with 4-H programs. Eight (five in focus group one and two in focus group two) of the 20 participants reported a close personal connection to someone on the autism spectrum (2- immediate family, 4-extended family, 2-friends).

### Focus Group Procedures

All research was conducted with approval from the Purdue University Institutional Review Board. The two individual focus groups (10 participants per group) took place at the Fall Youth Staff Conference for Extension Educators in September of 2019. Focus group questions were developed by a team consisting of two faculty members, four Extension Educators, and one graduate student. The semi-structured focus groups consisted of ten participants each, the moderator (CM) and the assistant moderator (VP). Moderators were both female with expertise in autism and intervention services for children on the autism spectrum, but no direct experience with 4-H. The moderator introduced the purpose of the study and participants received folders with consent forms to read and sign. The participants also filled out a brief survey. During the focus groups, participants were asked open-ended questions [see Table 1] and had space to share and discuss their opinions and experiences. In addition to questions in the guide, moderators used probes to clarify (e.g., “can you describe a little bit more..;” “What do you mean by that?”), to expand perspectives [e.g., “any on this side of the table?” “So I haven't heard from (Name), is there anything that you've…”], and to amplify responses (e.g., “You have touched a little bit on this before, but I would love to hear a little bit more about the types of supports and strategies you implemented to help students on the spectrum?”; “So do you have ideas about what kinds of formats would be more acceptable?”). Sessions were each 1 hour with 10 minutes for introductions and consent and ~50 minutes for questions (51.51 and 49.47). All participants contributed to the discussion. After the focus group, the moderator and assistant moderator held a debriefing session. Each of the focus groups was audio and video recorded. Recordings were later transcribed using Nvivo software.

**Table 1:** Research questions and corresponding focus group questions.

**Research question**	**Focus group question**
1.What are the barriers to enrollment in 4-H for youth on the spectrum	a) How did the youth with autism first get involved with 4-H
	b) What barriers, if any, do you see for students with autism who want to participate in 4-H but aren't already involved?
2. What types of training resources are available to Extension Educators within 4-H to create inclusive programming	a) What training did you receive on inclusion for students with autism?
3.What supports and strategies do Extension Educators actively use to support youth on the spectrum within their programs?	a) What types of supports or strategies have you implemented to support students with autism?
4.What challenges do Extension Educators and youth face related to inclusion?	a) As an Extension educator, what are the challenges you face when engaging students with autism?
	b) What do you see as the biggest challenges for students with autism who participate in 4-H?
5.What types of new strategies and resources would help to improve inclusion?	a) In what areas, if any, do you feel you need more support or resources?

### Analytic Plan

Our team used techniques from grounded theory (25, 26) and thematic analysis (27) in our analysis process. The initial phase was an iterative process where each team member engaged in open coding of the transcripts independently to develop a coding framework/codebook based on our research questions and the transcripts that resulted in both structural and data-driven themes. The team at this phase included two faculty members, two extension educators, one graduate student, and two undergraduate students. During this phase, team members met to discuss the identified codes and together, finalized a codebook. To build the codebook, individual codes were also initially grouped into larger categories or themes based on our research questions and those that emerged from the data (27). For each code, example quotes from within the data that best illustrated that code were also selected. This process allowed us to include themes we anticipated based on our research objectives and focus group guide but also allowed for openness to capture relevant themes that emerged during the coding process. In the second phase, individual team members (3 undergraduate students, 1 graduate student) independently coded each transcript within Nvivo. A larger team (all coders and two faculty members) then met to review the transcript coding line by line and discuss any coding discrepancies. Discrepancies were resolved via consensus. After consensus coding, a constant comparative approach was used to identify subthemes (26). In the final stage, axial coding was performed to link overlapping categories, reduce the final number of categories, and develop our final themes (25).

## Results

Five main themes emerged that directly addressed our research questions: (1) enrollment process and barriers; (2) Extension Educator challenges; (3) youth challenges; (4) active strategies; and (5) new supports and resources. Two data-driven themes were also identified that would potentially impact the development of new resources: (1) benefits; and (2) Extension Educator perspectives. Each theme and associated subtheme are described below with representative quotes. Themes are outlined in Table 2 and additional quotes are also reported in Supplementary Table 1.

**Table 2:** Results: Themes and subthemes.

**Theme**	**Subtheme**
Enrollment process and barriers	Autistic participation
	Getting involved
	Awareness
	4-H culture
Benefits	Skill development
	Personal development
Educator perspectives	Personal attitudes
	Extension leadership
	Personal experience
Educator challenges	Personalized supports
	Parent communication
	Working with others
	Training gaps
Youth challenges	Accommodations
	Fair process
	Peer issues
Active strategies	Individualizing
	Mentor mentoring
	Peer mentoring
	Outreach
New supports and resources	Increase enrollment
	New Strategies
	Resource portfolio
	Connections with parents
	Flexibility
	Mentoring
	New Trainings
	New Content
	Train-the-trainer
	Stakeholder Involvement

### Enrollment Process

During discussions of the enrollment process for families, participants described the types of programs and frequency with which they encountered youth on the autism spectrum within 4-H. They also described facilitators and barriers to enrollment.

#### Autistic Participation

All participating educators reported youth on the autism spectrum participating in their programs. The frequency varied from one youth member to several youth members over the years. As noted by one of the participants “We have had kids that have been involved in virtually every project.” Commonly reported programs were 4-H Camp, 4-H Academy, and a wide range of 4-H clubs (e.g., robotics, alpacas, arts and crafts).

#### Getting Involved

Extension Educators described several aspects of the enrollment process that facilitated getting youth on the autism spectrum enrolled in 4-H programs. Many Extension Educators mentioned that the youth on the autism spectrum within their programs came, “always from a family tradition.” This prior familiarity with the program made parents more likely to encourage their children to enroll as well. Several Extension Educators emphasized the importance of communication with new families when they described conversations that were necessary to make families feel comfortable enrolling. Educators and club leaders with personal experiences related to children with disabilities were also a draw for families to join. “I said you know you're, you're coming to a group of people that not only know how to do this but live it every day.” For the youth, Extension Educators mentioned that finding or creating clubs that aligned with their interests was a big draw for getting new 4-H members enrolled.

#### Awareness

The first major barrier related to enrollment was general awareness related to 4-H. Extension Educators reported talking to parents who had never heard of 4-H or were not aware that it was an inclusive program where children with disabilities “aren't treated differently.” In addition, knowing what's available and how to find the best fit could be a challenge. “Because I mean we have like 13 clubs you can pick from. So, how do know unless you're in dog obedience or you're in horse and pony, how do know what would be best for my kid to join?” Extension Educators also reported minimal referrals from schools or other service providers, indicating a lack of community awareness of 4-H as an option for youth on the autism spectrum.

#### 4-H Culture

Participants shared that being new to the 4-H environment and reading through documents, manuals, and rules books that families receive might be “like navigating a whole other language.” They also shared that all information is in written form, and therefore, the amount of reading required to learn about 4-H may be discouraging to families. The same difficulty might apply to the recruitment materials as it might be a challenge to communicate the benefits of 4-H programs when people do not want to read through the materials.

### Benefits

Although there were no specific probes related to benefits for youth participating in 4-H programs, positive aspects of the programs were a frequent theme within the discussion related to inclusion. This theme highlighted the potential impact of 4-H programs if they were able to reach more youth and improve their inclusive practices. Across both groups, participants shared that youth achieved the general goals of 4-H program related to practical and life skill development.

#### Practical Skill Development

As expected for all participants of 4-H programs, Extension Educators described how youth on the autism spectrum develop valuable practical skills through 4-H participation.

… he just developed a really close relationship with animals through the 4-H program and um you know could communicate with them in a way that was um amazing to watch and that was kinda his thing and he learned everything you know needed to know about those animals and um like you said was just able to really grow in his ability to communicate and um be in front of people.

Educators reported a wide range of skills including leadership, demonstration, independence, and communication, as well as skills specific to program topics (e.g., animal care).

#### Life Skill Development

Beyond the skills taught within the 4-H curriculum, Extension Educators conveyed that participation of youth on the autism spectrum often resulted in great personal development. “She had told me she always wanted to be a teacher, but she never had the confidence and when 4-H had the confidence in her to lead the robotics club, it gave her confidence uh confidence she had never experienced.” Some participants observed that youth on the autism spectrum developed their confidence to such a level that they took on the leadership roles (e.g., 4-H representative, liaison, and counselor).

### Youth Challenges

Moderators asked participants to share any challenges they observed youth on the autism spectrum face while participating in programs. Extension Educators' challenges related to specific contexts (fair process, peers) and also general issues related to accessing appropriate accommodations.

#### Accommodations

Youth on the autism spectrum come to 4-H needing additional supports that are specific and varied. The participants discussed examples of youth facing difficulties when those needs were not met.

We make sure the counselors know what the schedule is and what's happening all day, but we don't always make sure that those campers know what's gonna happen throughout the day. And I know [name], that was his anxiety with camp was like not knowing what was gonna happen.

Additional examples included youth with minimal verbal abilities who struggled with communication and the daunting amount of paperwork required in some programs. These examples highlighted the need for more flexibility and knowledge on the behalf of 4-H.

#### Fair Process

The most challenging context for youth on the autism spectrum was overwhelmingly the 4-H fairs. Youth often struggled to adhere to strict rules during judging and deviations from 4-H traditions were often met with resistance by fair organizers. Together this resulted in exclusion from activities or misjudgment of that youth's efforts and abilities. “When it–at the judging, ask my child a question the clock's turning and yet he has a problem with communication processing time. And the judge doesn't think he knows it, so he moves on to something else.”

The venue presents additional challenges, particularly for youth with sensory sensitivities.

It's not mandatory that you come to the judging but of course it's encouraged. And I know there are some who just don't come. Mom comes drops the project off and leaves. And so that 4-'Her's missing out on the opportunity that everyone else is getting because it's, it's just too overloading for them.

Overall the combination of the sensory intensity of the venue and difficulties interacting with unfamiliar adults make the fair process particularly difficult for youth on the autism spectrum.

#### Peer Issues

Participants described difficulties between youth on the spectrum and their neurotypical peers.

And so you know, it's hard to take 300 kids at camp and educate them. We try to do a good job with our counselors and our junior staff but sometimes kids don't understand why this person's a little different and can be, can be cruel.

Educators identified that often these challenges arise from a lack of awareness and acceptance on the part of the neurotypical youth that requires additional education to address.

### Extension Educator Perspectives

Throughout the focus groups, although not directly asked, participants revealed their perspectives, attitudes, and experiences that influenced their implementation of inclusive practices within their programs.

#### Personal attitudes

There was strong agreement across both focus groups that inclusion is an important aspect of 4-H. “…we, I think as educators, love the program we are in so much that we want to go to the nth degree to be inclusive and make it inclusive for every kid.” Many educators indicated the importance of not identifying children to be “different,” so as not to single out children with disabilities.

And I think that's one reason why 4-H is appealing to parents who are familiar with the program, because they aren't treated differently. They get the same public recognition [Yes] that all of the other 4-Hers get. [Mhmm] Without them physically standing out [Out as] or being different [being - yes!], yes.

Moreover, the participants shared their openness and desire to receive more training and resources on inclusion and including youth on the autism spectrum in their programs together with resources on how to support club leaders/volunteers, counselors, and judges, and lead effective communication with families.

#### Extension Leadership

All of the participants expressed receiving the overall message from 4-H leadership to be inclusive and support inclusive practices.

I mean we talk about inclusion all the time, but we don't specifically say include the people with autism or include this group it's just kinda a general everybody is welcome everybody should be included.

This was echoed frequently in emphasis that all youth are not to be “identified as different” within 4-H. However, Educators conveyed a desire for more support from Extension leadership in the form of increased practical training on inclusion.

#### Personal Experience

The participants reported the use of their own experiences interacting with youth on the autism spectrum as a resource to help guide other educators, club leaders, and volunteers when having youth on the autism spectrum in their programs. “So, umm being able to, to understand what their fears are. Having those conversations with some of my co-workers, you know, through the years that I've dealt with that gives us the resources to, you know, answer those questions.” The majority of these experiences originated from (a) being a parent of a child with a disability or being in a close relationship with them, (b) having previous experience of working with a child with a disability in their programs, and (c) having previous experience of working with a child with a disability out of 4-H (special ed. teachers, teachers). Participants reflected on key aspects that they learned from their own previous experiences and interactions when working with families with youth on the autism spectrum.

### Extension Educator Challenges

Researchers prompted participants to describe any specific challenges they faced in providing inclusive programming for youth on the spectrum. They shared several areas where they struggled with youth and with others important for the support of 4-H programs.

#### Personalized Supports

Participants recognized the importance of meeting the individual needs of the youth in their programs but sometimes struggled to create personalized supports for youth on the h autism spectrum.

I think there's so much diversity as far as the-the range of what to expect [Hmm] that it really is difficult. You really have to get to know that child and talk to the parents in order to find out what, what are the needs that I need to address. You know, that's my biggest challenge…

This was echoed by another participant.

…finding a way to get her to come to those things and get out of the house or stay longer, don't just come for show your goat and go home, but stay and engage and you know different things we're doing throughout fair week or camps or anything. That might be, that's a challenge for me…

The difficulty of developing and implementing individualized supports or accommodations to maximize youth participation spanned across all 4-H contexts.

#### Parent Communication

A large factor contributing to being able to provide appropriate supports across all contexts was communicating with parents of youth on the autism spectrum. Some frustration with communication came from not knowing how to facilitate difficult conversations.

And, and if we had like-we need to-I think, have more training on. You know. I don't know what how to handle a parent who doesn't want-who doesn't want to tell the educator to single a person out.

Breakdown of communication between parents and Extension Educators often intensifies the challenge of providing supports.

#### Working With Others

Extension Educators provide programs with a team that may have members with different perspectives, attitudes, and skills. These differences sometimes impact their ability to provide inclusive programs for their youth. One particular context that came up frequently was working with judges during 4-H fair activities. The participants shared that judges often lack knowledge about the challenges that youth on the autism spectrum might encounter during the fair process, leading to resistance to providing accommodations.

When it–at the judging, ask my child a question the clock's turning and yet he has a problem with communication processing time. And the judge doesn't think he knows it, so he moves on to something else. [Yeah. Yeaaah] That-this really upsets me, that they don't understand processing time. The judges need to learn some of those simple facts. [Mmhmm. Yeah. I agree.].

The Extension Educators expressed that they often struggle to communicate expectations and accommodations appropriate for the individual child due to conflicts between expectations of the program, maintenance of youth confidentiality, and respect for youth independence.

#### Training Gaps

Although the culture of 4-H promotes inclusion, Educators mentioned difficulties actually implementing inclusive practices due to gaps in their skills. This also impacted their ability to support the volunteers who have direct contact with youth “…like with my volunteers now, they want to know what, they want to learn how to work with these kids. And that's something that I can't give them because I haven't been educated on it.” Additionally, Extension Educators shared their experience of having negative feedback from club leaders/volunteers and parents on providing accommodations for youth on the autism spectrum. “I think there needs to be some training on how do we. How do you get that message across? [Yeah] That - Yes, adaptations are okay.”

### Training

Researchers asked participants to describe the training or education they have received on inclusion of youth on the autism spectrum. Participants described the training they received within Extension and beyond as well as the training opportunities they offered their volunteers.

#### Extension Training

Across both focus groups, all participants agreed that they received minimal official training on inclusion practices and strategies for children with disabilities through Extension. The only available training opportunities mentioned by the participants were webinars and presentations available through 4-H conferences that provide an overview of inclusion. The participants shared: “They weren't autism-specific. So sometimes you can find like those little nuggets, but they're usually like a 45-min webinar and it maybe skims the surface.” Further “That's what I was gonna say it was kinda like [kinda touch the surface] yeah, brief, but it wasn't even really a training it was like be respectful of all human beings, be inclusive.” Some described creating their own additional training opportunities with support from Extension.

…we had our area retreat where all the 4-H educators got together and um. I was on the planning committee and we made it a priority to have someone come in and talk to us about working with youth on the autism spectrum.

#### Non-extension Training

Many of the participants in the focus groups reported receiving training outside of Extension through (a) Individual initiatives to educate themselves on how to work with children with disabilities. “I've also attended sessions at the Indiana Youth Institute conference. Again, not Extension sponsored but -umm, we can attend it”; (b) previous education received as teachers or in classes during their degrees in special education; (c) their initiatives to educate volunteers, counselors, and themselves “…we've had guest speakers on working with kids with disabilities including autism, so I guess we've learning along with them in that type of training that we've provided that's bee like 30–45 min…”; (d) sharing experiences and knowledge with other educators and transferring the knowledge to counselors and volunteers. “I've really learned a lot from my fellow Educators.” As these types of learning opportunities were not offered by Extension leadership, they required motivation and initiative from the Educators to pursue.

#### Volunteer Training

Extension Educators provide training for other people who also work to directly support 4-H programs. The training they reported providing included: (a) sharing personal experiences and expertise and (b) formal and informal training sessions from Educators or service providers.

So, I have brought in [community service provider] to do a training with my volunteers on inclusion. And they've done, I've had them come one time and it was, I think I had like thirty-five volunteers at it. So, it was highly attended, and it's something needed.

Overall, these formal sessions were typically short, but they were offered regularly and sometimes became a learning opportunity for Educators as well.

### Active Strategies

Extension Educators were actively working with youth on the spectrum within their 4-H programs. They describe several types of strategies they already used to promote inclusion.

#### Individualizing

Extension Educators described creating individualized supports that focus on the youth's specific needs vs. their label.” …so I think sometimes we didn't look at it as autistic. We just looked at what do we need to do to make this work.” Reported individual supports included accommodations like extra time, creating written documents in place of oral presentations, and presenting projects early, before the arrival of the audience. Financial support to cover fees was also provided by 4-H and community members.

#### Mentor Mentoring

Further, Extension Educators discussed how they prepare club leaders, volunteers, counselors, and judges for working with and providing accommodations for youth on the autism spectrum. “… and so it helps for me to say: ‘hey, here's what's coming, here's how we can help them out.” Their goal is to “set them up for success” to secure positive experiences. For example, “like behind the scenes like at a before a show or before a workshop or something just so everybody's clear that nobody's being rude or disrespectful or you know not ignoring you.”

#### Peer Mentoring

As shared by the majority of the participants, the use of peer-mentoring support strategies is very common in the 4-H environment, particularly in the camp setting. They shared “I had 4-H kids that that would bond with him and help him and do it you know, and he, he did better with them than mom standing around.” Participant responses highlighted the potential for peer mentoring when implemented with training and supports from Educators.

#### Outreach

To increase enrollment, Extension Educators shared several outreach strategies they use to help share the message of 4-H as a great opportunity for youth on the autism spectrum. These individual efforts included sharing through after-school programs, special education services, social media groups, and, the most common one, word of mouth. “You're never gonna go wrong with word of mouth [mhm], so um, I mean that's our best-is somebody telling how the 4-H program has influenced them and their children and been a positive impact.”

### New Supports, Resources

Participants were asked to share their input on what types of new resources, strategies, and supports could improve their ability to provide inclusive programming for youth on the spectrum.

#### Increase Enrollment

Extension Educators shared strategies they believed would help to increase enrollment of youth on the autism spectrum. The participants agreed on the need for a change in recruitment language to advertise what 4-H has to offer to everyone including families with children with special needs.

…he's like 80% of the parents are just gonna toss it cause they'll be like it's not really for my kid. Getting words on paper that will actually resonate with parents and youth on the autism spectrum or whatever population you're trying to reach that would make them say “nope 4-H is really for my kid and I do want to be here.”

They further suggested using examples and promoting how youth on the autism spectrum can enjoy 4-H by utilizing parents who have lived the situations, they shared:

It seems to be person to person, so we're trying to develop a network of parents talking to other parents about why-why do they have their children in 4-H. Spread that word with your circle of other parents and, and help them see where their child would fit in.

They also suggested creating other forms of recruitment and advertisement such as videos and brochures.

#### New Strategies

Across both focus groups, there were several suggestions for new strategies or expanded strategies that could help improve inclusion for youth on the autism spectrum.

##### Resource Portfolio

Educators shared that they would like to have a “portfolio” of accessible and appropriate accommodations, supports, and resources. Ideally, these resources would include materials for Extension Educators and also include materials they could share with volunteers and parents. “And if just more resources to give [volunteers] and to help them um work with the kids that cause like you said, [name], they have more direct contact than we do.” This portfolio could also include direct access to experts with both professional and personal experience.

…a m-mom or a dad or a guardian of some sort you know I can reach out to that I can ask those questions to and I guess have that honest conversation of this is what I'm thinking about doing. Is this inclusive or not inclusive in your mind?

Some suggestions that would fit within a new resource portfolio related to creating sensory-friendly environments.

Where in our buildings are there space for them to go where they can just have their moment and refocus and come back? We also need to think about-just those learning environments. The lighting that we have [mhm], and things you know, you know those are things we need to think about and those are things we need to be trained on and then be able to take back to our volunteers. And you know, just make them aware and do their best to accommodate those possible.

Ideally, this would be a resource that Educators could access as needed and continue to build.

##### Connections With Parents

Multiple participants mentioned the need to connect more with parents through spending time in open conversation to assess youth needs. In addition to conversations with parents, participants suggested being more “proactive than reactive” by clearly stating in all early communications with parents that 4-H is inclusive and being open to communication about individual youth needs. One Extension Educator also emphasized the importance of communicating with each parent because of the range of unique experiences and needs.

I think that most of the parents would be more than willing to share their story and to share um their needs, but what you're going to get from one parent is their specific experience. So it's really important to um gather more.

Increasing communication with parents could help prepare youth for new situations by providing them and their families with detailed information about what to expect.

##### Flexibility

In the context of talking about 4-H-related paperwork requirements, one Educator suggested a need to change program expectations. “But just like as a program we, that are tradition or that we always do. Just making sure that we know like it's OK if it's not done the way we think it's going to be done.” This would be a shift toward more flexibility in the traditional 4-H requirements.

##### Mentoring

Participants identified peer mentors as an underutilized resource. As opposed to an adult telling them “what do and how to do it,” youth-to-youth support has the potential to increase engagement. Another participant stated, “We have to have volunteers who understand [Mmhmm] it who can relay the message to the kids to help them understand.” Participants acknowledged that to better implement mentoring this would take support throughout the tiered leadership structure.

#### New Trainings

Extension Educators provided several recommendations for new trainings related to inclusion.

##### New Content

Participants expressed a need for more training focused on comprehensive education that addresses the benefits of inclusion for everyone involved in 4-H. Specific suggestions included tangible information about strategies and effective accommodations. Participants also discussed the need for training, with practice opportunities, focused on strategies for communication with parents, youth, and others associated with 4-H (e.g., volunteers and judges). As one participant shared, “I think scenarios of role-playing would be helpful cause in any I don't care what we're doing, but the more practice we have at it the better.” So not only the amount of training but also the range of content and the delivery of that content needs to be addressed with new resources.

##### Train the Trainer

Extension Educators are responsible for supporting and training club leaders and other volunteers with 4-H, thus, several participants highlighted the need for training materials they could implement and share throughout their existing leadership structure. Materials that eventually reach club leaders, camp counselors, and other volunteers are essential because as one participant said, “The first face [youth] will likely see is their club leader and not ours.”

##### Stakeholder Involvement

Several participants expressed the importance of “lived” experience for people developing and delivering trainings. As one participant reflected,

Just in this room. We've all talked about kids that have been in our program maybe that parents are a good presenter, maybe that grandparent of a - of that child is also for 4-H [Mhmm] could be good presenters. But I think we really need presenters that understand what we do that understand the program and understand the good days and the challenging days. Because I think I think, that's where you walk away from that training feeling that you've got something that - that you can use on a day to day basis.

The inclusion of stakeholders who have experiences working and living with people with disabilities as well as knowledge of 4-H would enhance the impact and effectiveness of new trainings for Extension Educators and the families they serve.

## Discussion

The goals of this study were to identify barriers to enrollment, to understand the current challenges of Extension Educators and youth on the spectrum, and to inform the development of resources to improve access and inclusion for youth on the spectrum in 4-H. To accomplish these goals, two focus groups were conducted with Extension Educators who support 4-H programs throughout Indiana. Several areas of need, as well as the potential for new resources, emerged. Although the mission of 4-H as an inclusive program is promoted and there is great willingness from Extension Educators, there is a lack of effective training to prepare those supporting 4-H to deliver fully inclusive programing, which aligns with previous reports (28–31). Additional challenges, active strategies, and potential for new resources were identified.

Two major challenges related to enrollment were awareness and 4-H culture. Despite active strategies on the behalf of Educators that included connecting with new families through school services, social media, and word of mouth, Educators conveyed a lack of knowledge in the general community that 4-H is an inclusive program and that there is a wide range of learning opportunities available for youth. Participants also highlighted the daunting task of navigating the processes and procedures involved with 4-H, especially for families that are new to the program. These results indicate a need for messaging across all levels of 4-H to highlight the diversity of programs available and emphasize that all youth, including those with disabilities, are welcome to participate. Many Extension Educators emphasized the effectiveness of personal connections in encouraging new families to try 4-H; however, this strategy is concentrated and time-consuming. Building up the outreach network to maximize the reach of current 4-H families, school staff, and other service providers could help reach more new families. Additionally, 4-H programs may benefit from building in more support and flexibility during the onboarding process. As current numbers of youth with disabilities enrolled within 4-H are unknown, future studies would need to closely track enrollment numbers.

Once enrolled in the program, Educators noted that youth on the autism spectrum face specific challenges. The most common context for challenges noted was 4-H fairs. The sensory environment, social demands, and rigidity of the rules for participation create a difficult situation for youth to navigate, which all relate to common themes of environmental factors that hinder the participation of youth on the autism spectrum (32). This context highlights the need for individualized accommodations, increased flexibility, and better communication between parents, youth, and 4-H personnel.

Some similar needs emerged in the analysis of challenges specific to Extension Educators. In particular, communication with adults involved in 4-H and parents of youth was highlighted as creating difficulties in developing and supporting appropriate inclusive programming. Regular communications between parents and Extension Educators that provide space for all parents of youth participating in 4-H to share information about the needs of their children, could help facilitate better communication without requiring parents to disclose their child's diagnosis (33).

The focus group data provided useful information about additional resources that could help improve the inclusiveness of 4-H programming. Extension Educator views reflected the consensus across many reports related to the inclusion of youth with disabilities, that there is a major need for more training and resources to effectively include youth with disabilities in 4-H programs (31). New materials to meet this need would best fit into the current model of 4-H if they were designed for Extension Educators as the primary consumer, which they would then share with volunteers and other 4-H supporters who work directly with youth. A train-the-trainer model would not only fit with the current 4-H structure but has also demonstrated effectiveness in disseminating evidence-based practices in community settings (34). Additional training resources could increase the number of Educators who provide training for club leaders and volunteers (18). Content and trainings should all be developed with input from people with direct personal experience with both 4-H and autism. Trainings and resources that are more generalized and not focused on the label of autism would align with the desire that youth not be singled out. This type of need-focused vs. specific label-focused content could also benefit children with other types of disabilities (e.g., ADHD), who are also impacted by the lack of training on disability inclusion (29).

### Limitations

Findings from this study should be reviewed in light of a few limitations. The focus groups included only Extension Educators. Additional challenges, needs, and ideas for new resources could be identified if the focus groups included additional stakeholder groups. Future work will include 4-H volunteers, parents, and youth perspectives. Additionally, some of the participating Extension Educators had close ties to people on the autism spectrum. This experience may have increased support for inclusive practices and also provided perspectives that are not reflective of the perspectives of those Educators without personal connections to autism. Focus group participants were also exclusively from Indiana. Although the basic structure of 4-H across the Unites states is similar, Extension, and subsequently 4-H, does have some state-level variations which may be noted if this study were to be replicated across states.

### Conclusions

The structure and purpose of 4-H naturally lends itself to the inclusion of all youth, including those on the autism spectrum. Thus, 4-H has the potential to provide opportunities for skill-building, social-communication practice, and the development of friendships for youth on the autism spectrum within a natural environment. Maximizing this experience necessitates additional training, increased channels of communication between parents and extension educators, and identification of mechanisms for the provision of individualized supports and accommodations to promote positive experiences. With increased support, 4-H and programs like it could address the service needs of youth on the spectrum, particularly in rural areas. As identified in this analysis, perspectives and involvement of stakeholders with personal connections to autism and 4-H are needed to develop new resources that will address youth needs and maximize the positive impact of 4-H.

## Data Availability Statement

The raw data supporting the conclusions of this article will be made available by the authors, without undue reservation.

## Ethics Statement

The studies involving human participants were reviewed and approved by Purdue University Institutional Review Board. The patients/participants provided their written informed consent to participate in this study.

## Author Contributions

CM is the principal investigator and lead author. CM and VP conducted the focus groups. CM, RM, VP, EC, and AM contributed to the data analysis process. All authors contributed to the writing of the manuscript.

## Funding

This study was funded by the Agricultural Science and Extension for Economic Development (AgSEED) program at Purdue University's College of Agriculture. CM received support from the National Center for Advancing Translational Sciences (KL2TR002530 and UL1 TROO2529).

## Conflict of Interest

The authors declare that the research was conducted in the absence of any commercial or financial relationships that could be construed as a potential conflict of interest.

## Publisher's Note

All claims expressed in this article are solely those of the authors and do not necessarily represent those of their affiliated organizations, or those of the publisher, the editors and the reviewers. Any product that may be evaluated in this article, or claim that may be made by its manufacturer, is not guaranteed or endorsed by the publisher.
