# Supplementary material for: “Setting Them Up for Success”: Including Youth on the Autism Spectrum in 4-H
Source: Front Psychiatry. 2022 May 31;13:913600. doi: 10.3389/fpsyt.2022.913600 (PMC9193588; doi:10.3389/fpsyt.2022.913600)
Supplement: Supplementary file 1 [file Table_1.DOCX]

Supplementary Material

# Supplementary Tables

| Table S1. Themes, subthemes, and additional quotes. | | |
| --- | --- | --- |
| Theme | Subtheme | Quotes |
| Autism Participation | Frequency | “Um, I've had a couple different youths that I've worked with that um are on the spectrum. I've had kids go to camp um I've had a couple kids in the robotics program, I would say that's where has been my biggest interaction with um those youth and I've had some kids go to round-up as well.” |
|  | Program types | “I've had one participate in public speaking at the county level. I have one currently who shows poultry and does photography and comes to a lot of sport clubs and has recently joined, he's just now eligible for junior leaders so. Mom was nervous. I said he’ll be just fine. So.” |
| Enrollment Process and Barriers | Getting Involved | “I was gonna say parents. Parents was a big one for me. [others agreeing] Their parents pushed them to try it. Uh, some of them were 4-Hers themselves, some of them came to our open house, um some of them knew people already in 4-H and was kinda friends and family.” |
|  | Awareness | “Often times it's hard to hook people who maybe haven't had a long history of involvement…” |
|  | 4-H Culture | “One barrier for all kids and families who are brand new to 4-H is the massive culture that 4-H is. It's like navigating a whole other language [mhm], so that and I - for kids with any ability and it's not just signing up for a rehearsal or a practice and then the games are this day and then you just know that's when you show up. So many of us are giving them 85-100 pages when they walk in the door and they're like here's the rules book. That's a huge barrier for a lot of people [mhm].” |
| Benefits | Skill Development | “And so, for I mean, by the end of 10 years I had a 4-Hr who could get up and I mean, he was still nervous and he, but he could give the demonstration. And we've started with a he, he wrote something and me or one of the other adults read it. That was, we made that accommodation. There was no way he, it was enough for him to get up from the corner and come sit up here in front of everybody.” |
|  | Personal Development | “In addition to what everybody else said is that, you know. We've had several members through the years. Now when I go to camp. We've had some of them actually serve in a general leadership role over the years.” |
| Youth Challenges | Accommodations | “What I've noticed a lot is most of the kids really struggle with communication. [Mmhmm] And I even talked to [name] about think if we could do something where. Like my son, he is, it's not that he's non - not I mean, he's verbal. But it's hard to understand him. And he, he gets caught in the track. You were talking about getting in the loop. [Mmhmm] Of you know, if we could do an iPad with some kind of an upper like a pictograph. His communication is really difficult and some kids are so shy they won't communicate. [Mmhmm] That's a real hurdle for, for my kids and in my program.” |
|  | Fair Process | “I didn't know that that wasn't going to be OK with him and maybe mom didn't know either but. Put on the spot like that it was not -it was kind of an awful situation.” |
|  | Peer Issues | “But I have also seen, as [name] says. There's been some kids who don't have that knowledge. They don't understand they just think they're weird” |
| Educator Perspectives | Personal attitudes | “Some of that training comes back to your desire to be what the program needs to be with every individual. Regardless of [Yeah, Mmhmm] whether they're late, you know, have autism, or Downs or special needs.” |
|  | Extension Leadership | “Well, long - long time ago they came out with a little bitty brown binder [which I have still] from like nineteen ninety something. That's the only thing I've ever seen [I have that]. That encouragement to be inclusive.” |
|  | Personal Experience | “I'm a parent of a child so I've lived the experience but honestly there's not been a lot of education that anybody's really offered me either as a parent.” |
| Educator Challenges | Personalized Supports | “I don't know what how to handle a parent who doesn't want - who doesn't want to tell the educator to single a person out, but at the same time - no you can't go back there. So, we're stuck right here at the tables and I have to. We have to accommodate really quickly. And yeah it blows up because - [Mmhm] it just does.” |
|  | Parent Communication | “And sometimes I think a parent gets frustrated - I can remember a parent getting frustrated with, with us in the office. But she never came in on the front end. And I was frustrated, the fact that why didn't you - well, maybe you didn't reach out to me, so then you get this. So it's kind of like an extension learning, who, who needs to kind of, you know, [Yeah] I guess facilitate that conversation” |
|  | Working with Others | “I think too, another challenge is this is when we talk about judges. But I also have had a lot of pushback from volunteers about - If you make adaptations for these kids. Well that's not fair. |
|  | Training Gaps | “I don't think that I have received any formal training, it's been more of what I've had to learn on my own as far as how to serve the youth and usually that come from conversation with the parents.” |
| Training | Extension Training | “We talk about inclusion all the time, but we don't specifically say include the people with autism or include this group it's just kinda a general everybody is welcome everybody should be included.” |
|  | Non-Extension Training | “So, the only training is back when I was getting my degree in college and I don't remember that part. [Mmhmm - laughter] It wasn't that long thought, but I don't remember that far back.” |
|  | Volunteer Training | “Umm, I have made an attempt to do some on my own and I always have a lesson with my volunteers saying: "Ok, you know my son, [name], [laughs] this is a typical [laughs] and - and tried to talk to them about how to deal with, with the kids. Because sometimes it is a little unusual.” |
| Active Strategies | Individualizing | “One thing that sticks out to me that I read a while ago that if you've worked with one kid on the autism spectrum you've worked with one kid on the autism spectrum.” |
|  | Mentor Mentoring | “I have a club leader who is very passionate about working with youth with autism…We're looking at different ways to, to help implement some things” |
|  | Peer Mentoring | “I have used older kids or peers of kids that, I have this one with Asperger's and of course that was a special relationship already, I mean otherwise it wouldn't work. But they kind of were there to help be, the go, between to help when this child got overwhelmed and stressed and that was very helpful.” |
|  | Outreach | “I've reached out to all the special needs special ed. departments in our county, just to let them know how positive the program can be.” |
| New Supports and Resources | Increase Enrollment | “I think it would be great to have some sort of brochure or some sort of literature or something that we could give to families that maybe we could promote you know in the special education program at the schools and things like that to let them know that 4-H really is for all and that they shouldn't feel excluded just by what they see at the fair or what they see you know in the paper that we're open to everyone.” |
|  | New Strategies | “Having like almost like a pool of ways to make something more accessible to pull from. So that you're not always like having to come up with like a brand new situation. But you know, so like, we know this worked, this worked two years ago, this worked 10 years ago, and this worked with somebody else recently. Would any of these work for you, or would you like to try any of those? Rather than like, OK let's sit and stare at each other until one of us thinks of something that would work.” |
|  | New Trainings | “Just how to have those conversations to make a parent comfortable and to make sure that I know what their child needs and then, how to convey that effectively to volunteers.” |
